# Clinical benefits and complication profile of IL-23 inhibitors in patients with psoriatic arthritis: a systematic review and meta-analysis

**DOI:** 10.3389/fphar.2025.1669786

**Published:** 2025-11-06

**Authors:** Jingxin Zeng, Ling Lin, Wei Li, Xinjing Gao, Qian Li, Xin Zhou, Weiyu Liu, Xuelian Zhong, Yunqing Yang, Xibao Zhang, Quan Luo

**Affiliations:** 1 Department of Dermatology, Guangdong Second Provincial General Hospital, Guangzhou, Guangdong, China; 2 Department of Dermatology, Guangzhou Dermatology Hospital, Guangzhou, Guangdong, China; 3 Department of Dermatology, Guangzhou Women and Children’s Medical Center, Guangzhou Medical University, Guangzhou, Guangdong, China

**Keywords:** psoriatic arthritis, IL-23 inhibitors, randomized controlled trials, ACR response, PASI90, minimal disease activity

## Abstract

**Background:**

Psoriatic arthritis (PsA) is a chronic inflammatory disease with heterogeneous manifestations affecting both joints and skin. Interleukin-23 (IL-23) plays a central role in the Th17-mediated inflammatory pathway implicated in PsA pathogenesis. This meta-analysis aimed to evaluate the clinical efficacy and safety profile of IL-23 inhibitors in the treatment of PsA.

**Methods:**

A systematic literature search was conducted across PubMed, Embase, Web of Science, and Cochrane Library up to June 30, 2025, adhering to PRISMA guidelines. Randomized controlled trials (RCTs) involving adult Psoriatic Arthritis (PsA) patients treated with IL-23 inhibitors versus placebo were included. Key outcomes analyzed included American College of Rheumatology (ACR) 20, 50, and 70 responses, Psoriasis Area and Severity Index (PASI) 90, minimal disease activity (MDA), and enthesitis and dactylitis resolution.

**Results:**

Six RCTs involving IL-23 inhibitors were included. IL-23 inhibitors significantly improved ACR20 (RR = 1.86; 95% CI: 1.69–2.05), ACR50 (RR = 2.75; 95% CI: 2.31–3.29), and ACR70 (RR = 3.06; 95% CI: 2.29–4.10) responses. Skin clearance (PASI90) was markedly higher (RR = 5.98; 95% CI: 4.68–7.64). IL-23 inhibition also resulted in superior MDA (RR = 2.85; 95% CI: 2.30–3.54), and better resolution of enthesitis (RR = 1.46; 95% CI: 1.29–1.64) and dactylitis (RR = 1.39; 95% CI: 1.20–1.61). Publication bias was not detected.

**Conclusion:**

IL-23 inhibitors are effective in improving musculoskeletal and dermatologic outcomes in PsA, supporting their role in comprehensive treatment strategies. Further long-term comparative studies are needed.

**Systematic Review Registration:**

clinicaltrials.gov, identifier CRD420251169783.

## 1 Introduction

Psoriatic arthritis (PsA) is a chronic, immune-mediated inflammatory arthropathy that affects up to 30% of individuals with psoriasis, characterized by peripheral arthritis, axial disease, dactylitis, enthesitis, and skin and nail involvement. The pathogenesis of PsA is multifactorial, involving complex genetic predispositions, environmental influences, and dysregulation of the innate and adaptive immune systems. Among the various cytokines implicated in the immunopathogenesis of PsA, interleukin-23 (IL-23) has emerged as a critical upstream regulator, playing a pivotal role in the differentiation and maintenance of pathogenic Th17 cells, which contribute to synovial inflammation, bone erosion, and entheseal damage.

The IL-23/IL-17 axis has been increasingly recognized as a key therapeutic target in spondyloarthropathies, including PsA. IL-23 is a heterodimeric cytokine composed of a p19 and a p40 subunit, the latter shared with IL-12. It exerts its pro-inflammatory effects through the activation of the Janus kinase-signal transducer and activator of transcription (JAK-STAT) signaling pathway, promoting the production of downstream effector cytokines such as IL-17A, IL-17F, and IL-22. Targeted inhibition of IL-23 via monoclonal antibodies against the p19 subunit—such as guselkumab, tildrakizumab, and risankizumab—has demonstrated substantial efficacy in treating moderate to severe plaque psoriasis (Kerschbaumer et al., 2024; Thomas et al., 2024). However, the therapeutic value and safety profile of these agents in PsA require further synthesis and systematic evaluation. Currently, the therapeutic landscape for PsA includes nonsteroidal anti-inflammatory drugs (NSAIDs), conventional synthetic disease-modifying antirheumatic drugs (csDMARDs), biologic agents targeting tumor necrosis factor-alpha (TNF-α), IL-12/23 (e.g., ustekinumab), IL-17 (e.g., secukinumab and ixekizumab), and Janus kinase inhibitors. While TNF inhibitors have long been the mainstay of biologic therapy, limitations such as primary and secondary non-response, adverse events, and contraindications necessitate alternative treatment options. Given the heterogeneity of PsA manifestations and patient responses, there is growing interest in evaluating the efficacy and tolerability of IL-23 inhibitors as a newer class of biologic agents (Neurath et al., 2024; Jiang et al., 2023; Loft et al., 2020).

To address these knowledge gaps, we conducted a comprehensive systematic review and meta-analysis of the available literature to assess the clinical benefits and complication profile of IL-23 inhibitors in patients with psoriatic arthritis. Through this analysis, we seek to provide a consolidated evidence base to inform clinical decision-making and guide future research on IL-23-targeted therapies in PsA management.

## 2 Methods

### 2.1 Search strategy

This study was conducted in accordance with the Preferred Reporting Items for Systematic Reviews and Meta-Analyses (PRISMA) guidelines (Page et al., 2021). A systematic literature search was carried out across four major electronic databases: PubMed, Embase, Web of Science, and The Cochrane Library, covering all publications from the date of database inception to June 30, 2025. There were no restrictions on language, and studies published in non-English languages were considered if an English abstract was available. To ensure completeness, reference lists of all included studies and relevant systematic reviews were manually screened for additional eligible publications. The full search strategies for each database are detailed in Supplementary Table S1. This study has been registered in the International Prospective Register of Systematic Reviews (PROSPERO) under the registration number CRD420251169783.

### 2.2 Inclusion criteria and exclusion criteria

Inclusion criteria: (1) the study population consisted of adult patients diagnosed with psoriatic arthritis based on established classification criteria (e.g., CASPAR criteria); (2) the intervention involved the use of IL-23 inhibitors; (3) Only randomized controlled trials (RCTs) were included; (4) relevant outcomes were reported, including clinical efficacy measures and/or safety outcomes; and (5) sufficient data were available to calculate effect sizes.

Studies were excluded if they met any of the following conditions: (1) the study was a review, meta-analysis, conference abstract without full-text, editorial, case report, or commentary; (2) the study did not report separate data on IL-23 inhibitors or combined them with other treatments in a way that prevented extraction of specific data; (3) the study population did not include patients with a confirmed diagnosis of psoriatic arthritis; or (4) the full text was unavailable or the outcome data were incomplete or irretrievable even after contact with authors.

### 2.3 Data extraction

Two reviewers independently screened the literature and extracted data in accordance with the predefined inclusion and exclusion criteria. Initial screening was performed by reviewing titles and abstracts to exclude studies that were clearly irrelevant. Full-text articles were then retrieved and reviewed in detail to determine final eligibility. Any discrepancies in study selection or data extraction were resolved through discussion, and if consensus could not be reached, a third reviewer was consulted for adjudication. Data were extracted using a standardized form, which included the following study characteristics and clinical variables: first author, year of publication, therapeutic regimen (including type and dosage of IL-23 inhibitor), average age of participants, number of male patients, duration of psoriatic arthritis (in years), baseline Psoriasis Area and Severity Index (PASI) score (0–72), with PASI90 defined as ≥90% improvement from baseline; swollen joint count; and tender joint count. All extracted data were cross-checked to ensure consistency and accuracy prior to analysis.

### 2.4 Quality assessment

The risk of bias of the included randomized controlled trials was independently assessed by two reviewers using the Cochrane Risk of Bias 2.0 (RoB 2.0) tool (Sterne et al., 2019). This tool evaluates potential bias across five domains: (1) bias arising from the randomization process, (2) bias due to deviations from intended interventions, (3) bias due to missing outcome data, (4) bias in measurement of the outcome, and (5) bias in selection of the reported result. Each domain was rated as “low risk,” “some concerns,” or “high risk” of bias, and an overall risk of bias judgment was assigned to each study accordingly. All assessments were cross-checked, and any discrepancies between the reviewers were resolved through discussion. If consensus could not be reached, a third reviewer was consulted for adjudication.

### 2.5 Statistical analyses

All statistical analyses were conducted using Review Manager (RevMan, version 5.4) and Stata (version 18.0; StataCorp, College Station, TX, United States). When studies reported medians, interquartile ranges, or standard errors instead of means and standard deviations, the SDs were estimated using established conversion formulas. Effect sizes were synthesized using either a fixed-effect model or a random-effects model, depending on the degree of heterogeneity observed across studies. Statistical heterogeneity was assessed using Cochran’s Q test and quantified with the I^2^ statistic. A p-value <0.10 or an I^2^ value greater than 50% was considered indicative of substantial heterogeneity, in which case a random-effects model was applied; otherwise, a fixed-effect model was used. Publication bias was assessed using Egger’s linear regression test, with a p-value <0.05 indicating significant small-study effects suggestive of publication bias. All statistical tests were two-sided, and a p-value <0.05 was considered statistically significant unless otherwise specified.

## 3 Results

### 3.1 Search results and study selection

**FIGURE 1:**
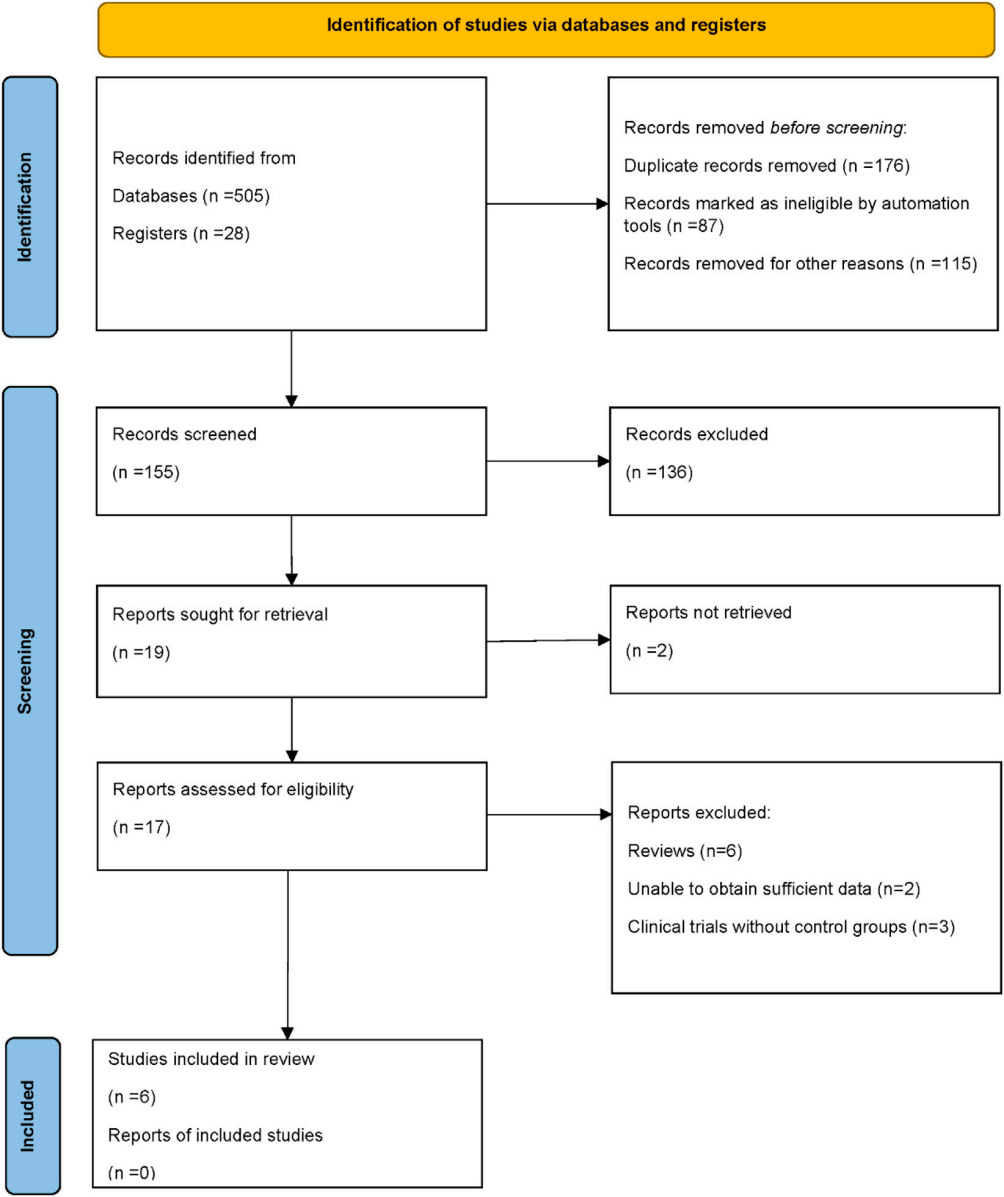
PRISMA flow diagram of study selection.

A total of 533 records were identified through database and register searches, including 505 records from electronic databases and 28 from trial registers. After removing 176 duplicate records, 87 records were excluded by automation tools, and 115 were excluded for other reasons. Ultimately, 155 records remained for title and abstract screening. Following the initial screening, 136 records were excluded due to irrelevance or failure to meet the inclusion criteria. The remaining 19 full-text reports were sought for retrieval, of which 2 could not be obtained. A total of 17 full-text articles were assessed for eligibility. Among them, 6 were excluded because they were review articles, 2 lacked sufficient data for analysis, and 3 were clinical trials without control groups. Finally, 6 studies met the inclusion criteria and were included in the qualitative and quantitative synthesis. A summary of the study selection process is presented in the PRISMA flow diagram (Deodhar et al., 2018; Mease et al., 2020; Coates et al., 2022; Kristensen et al., 2022; Östör et al., 2022; Mease et al., 2021) (Figure 1).

### 3.2 Study characteristics

A total of six RCTs were included in this meta-analysis, encompassing patients with active PsA who received IL-23 inhibitors or placebo. The studies evaluated three IL-23p19 inhibitors: guselkumab, risankizumab, and tildrakizumab. The mean age of participants ranged from 44 to 53 years across studies. The duration of PsA varied widely, with mean values ranging from 5.5 ± 5.9 to 8.7 ± 7.2 years. The baseline PASI scores ranged from 5.0 ± 6.5 to 13.6 ± 9.0, indicating a moderate to high degree of cutaneous disease severity among enrolled patients. Similarly, swollen joint count (0–66) ranged from 9.0 ± 6.0 to 13.6 ± 9.0, and tender joint count (0–68) ranged from 18.0 ± 11.0 to 22.8 ± 14.9, reflecting considerable musculoskeletal involvement (Table 1).

**TABLE 1:** Baseline clinical characteristics of patients in included studies.

Author	Year	Treatment group	Mean age (years)	PsA duration (years)	PASI score (0–72)	Swollen joint count (0–66)	Tender joint count (0–68)
Coates, L. C.	2022	Guselkumab 100 mg week 0, week 4, then every 8 weeks	49	8.3 ± 7.8	11.7 ± 11.9	10.0 ± 7.0	21.0 ± 13.0
		Placebo	49	8.7 ± 7.2	9.2 ± 9.4	10.0 ± 7.0	21.0 ± 13.0
Kristensen, L. E.	2022	Risankizumab 150 mg at weeks 0, 4 and 16	52	7.1 ± 7.0	10.9 ± 10.1	9.0 ± 6.0	18.0 ± 11.0
		Placebo	52	7.1 ± 7.7	10.9 ± 10.1	12.1 ± 7.8	20.8 ± 14.1
Ostor, A.	2022	Risankizumab 150 mg at weeks 0, 4 and 16	53	8.2 ± 8.2	7.7 ± 6.7	13.0 ± 8.7	22.8 ± 14.9
		Placebo	52	8.2 ± 8.3	10.0 ± 10.4	12.2 ± 8.0	20.5 ± 12.8
Mease, Philip J.	2021	Tildrakizumab 200 mg every 4 weeks	50	7.5 ± 8.5	8.4 ± 9.9	13.6 ± 9.0	22.3 ± 13.8
		Placebo	48	6.3 ± 6.1	5.0 ± 6.5	11.8 ± 9.8	19.7 ± 14.7
Mease, Philip J.	2020	Guselkumab 100 mg every 4 weeks	46	5.5 ± 5.9	10.8 ± 11.7	12.9 ± 7.8	22.4 ± 13.5
		Placebo	46	5.8 ± 5.6	9.3 ± 9.8	12.3 ± 6.9	21.6 ± 13.1
Deodhar, Atul	2018	Guselkumab 100 mg at week 0, week 4, and every 8 weeks	47	7.0 ± 7.2	12.0 ± 10.5	11.9 ± 7.6	20.7 ± 12.2
		Placebo	44	6.9 ± 7.2	9.9 ± 8.0	10.6 ± 7.5	20.1 ± 12.5

PsA, Psoriatic Arthritis; PASI, Psoriasis Area and Severity Index.

### 3.3 Results of quality assessment

**FIGURE 2:**
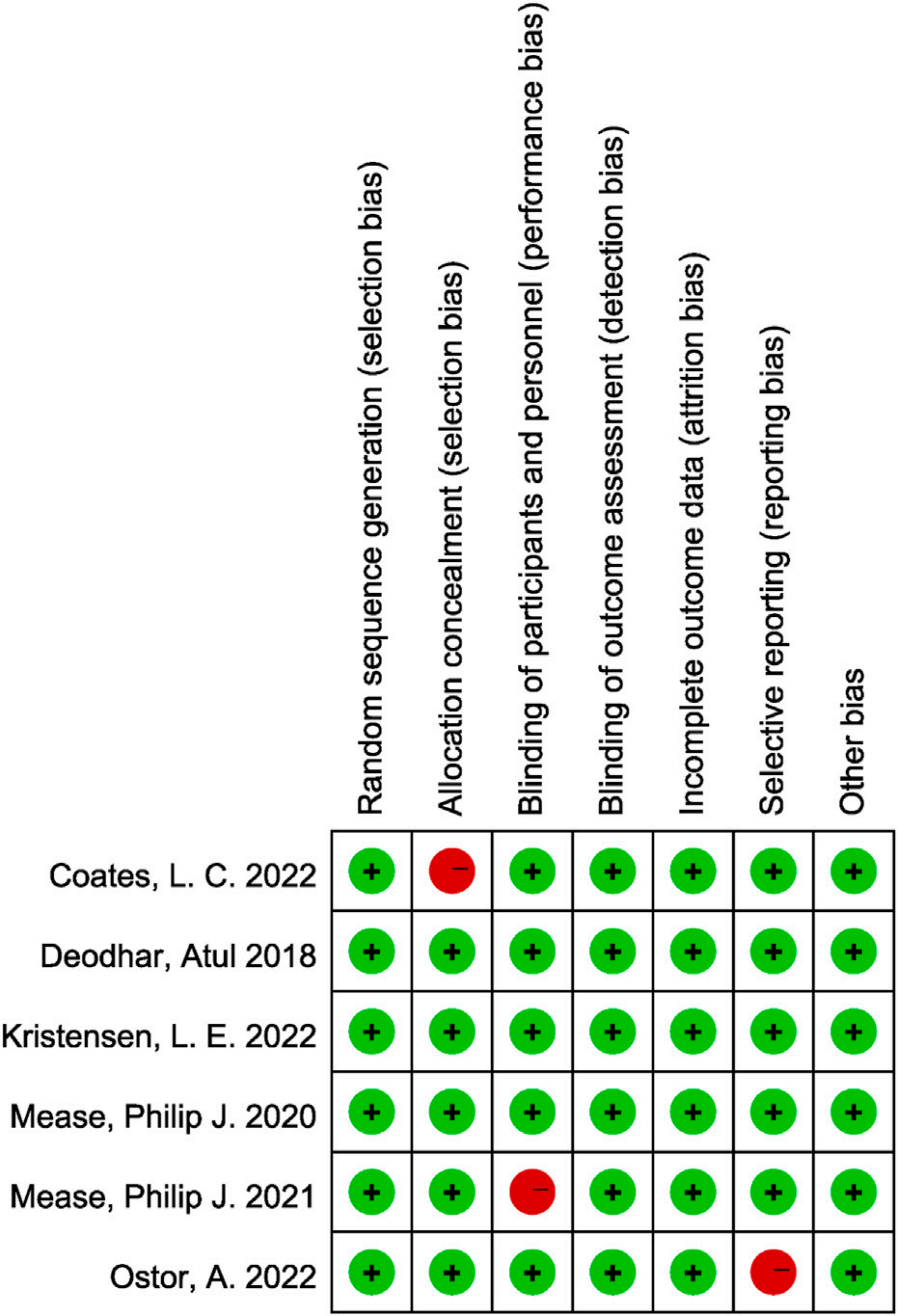
Risk of bias assessment of included studies using the Cochrane RoB 2.0 tool.

The methodological quality of the included randomized controlled trials was assessed using the Cochrane Risk of Bias tool. Overall, the studies demonstrated a low risk of bias across most domains. All trials adequately reported random sequence generation and blinding of participants, personnel, and outcome assessors. The majority of studies also showed low risk for incomplete outcome data, selective outcome reporting, and other sources of bias. However, two studies demonstrated a high risk of bias in allocation concealment, and one study exhibited a high risk of attrition bias due to incomplete outcome data. These limitations were not pervasive across the dataset and were considered unlikely to significantly compromise the overall validity of the findings. Taken together, the included trials were judged to be of generally high methodological quality, providing a reliable basis for the pooled quantitative analysis (Figure 2).

### 3.4 Efficacy outcomes: ACR and PASI responses

**FIGURE 3:**
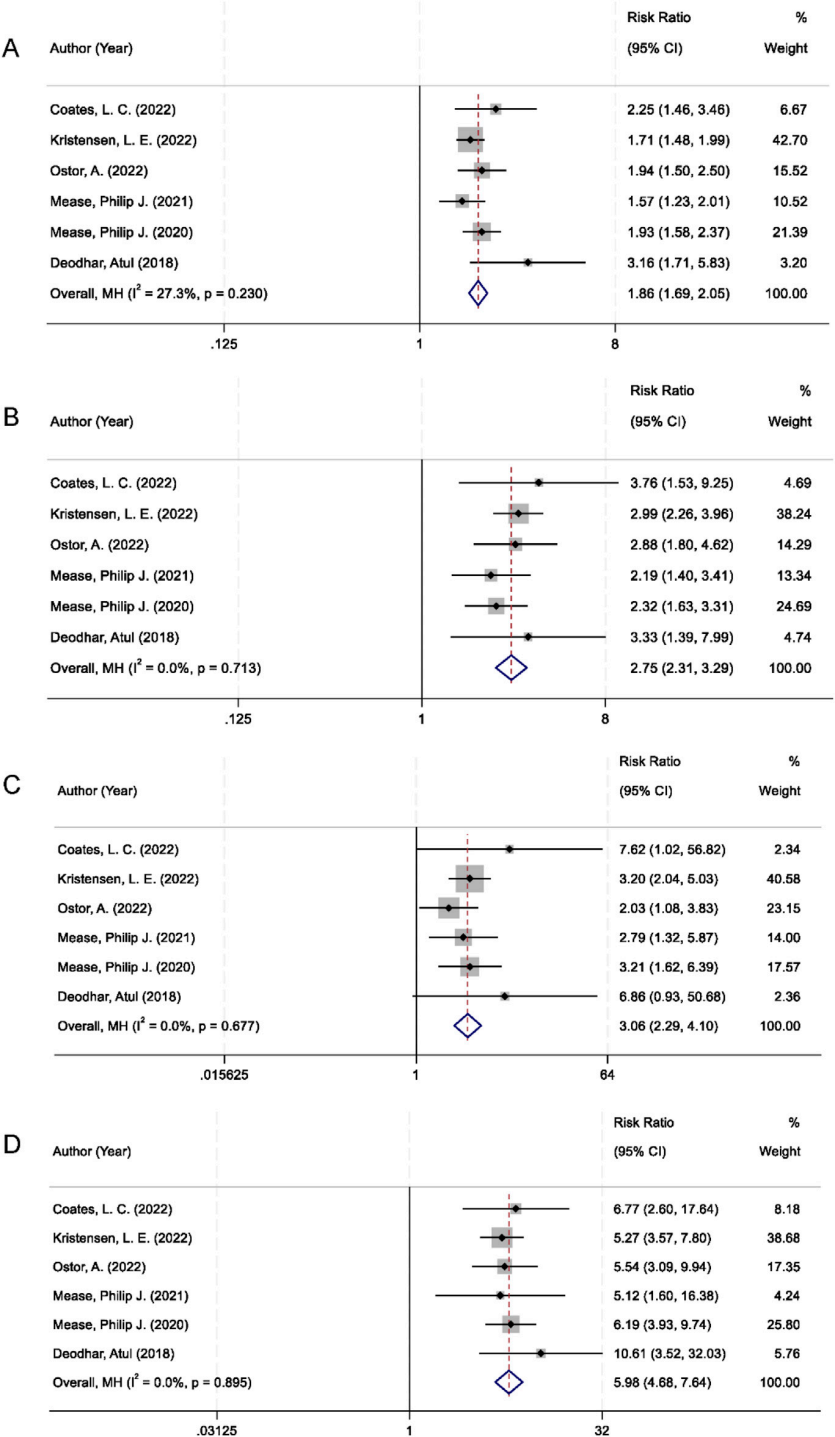
Forest plots comparing IL-23 inhibitors vs. placebo in PsA: **(A)** ACR20 response; **(B)** ACR50 response; **(C)** ACR70 response; **(D)** PASI90 response.

All six randomized controlled trials included in the analysis reported American College of Rheumatology (ACR) response rates at the 20%, 50%, and 70% improvement thresholds (ACR20, ACR50, and ACR70). The pooled analysis demonstrated that IL-23 inhibitors were significantly more effective than placebo in achieving ACR20 response. There was no significant between-study heterogeneity (I^2^ = 27.3%, p = 0.230), and the combined relative risk (RR) was 1.86 (95% confidence interval [CI]: 1.69–2.05; p < 0.001; Figure 3A). With respect to secondary outcomes, IL-23 inhibitor therapy also resulted in markedly higher ACR50 and ACR70 response rates compared to placebo. The pooled RR for ACR50 was 2.75 (95% CI: 2.31–3.29; p < 0.001), with no observed heterogeneity among studies (I^2^ = 0.0%; Figure 3B). Similarly, the ACR70 response was significantly elevated in the IL-23 inhibitor group (RR = 3.06; 95% CI: 2.29–4.10; p < 0.001), with no statistical heterogeneity detected (I^2^ = 0%; Figure 3C).

In addition to joint-related outcomes, skin symptom relief was evaluated using the Psoriasis Area and Severity Index 90 (PASI90), which indicates at least 90% improvement from baseline. Patients receiving IL-23 inhibitors were significantly more likely to achieve PASI90 compared to those in the placebo group. The pooled RR was 5.98 (95% CI: 4.68–7.64; p < 0.001), with no heterogeneity observed across studies (I^2^ = 0%; Figure 3D).

### 3.5 Minimal disease activity and resolution of enthesitis and dactylitis

**FIGURE 4:**
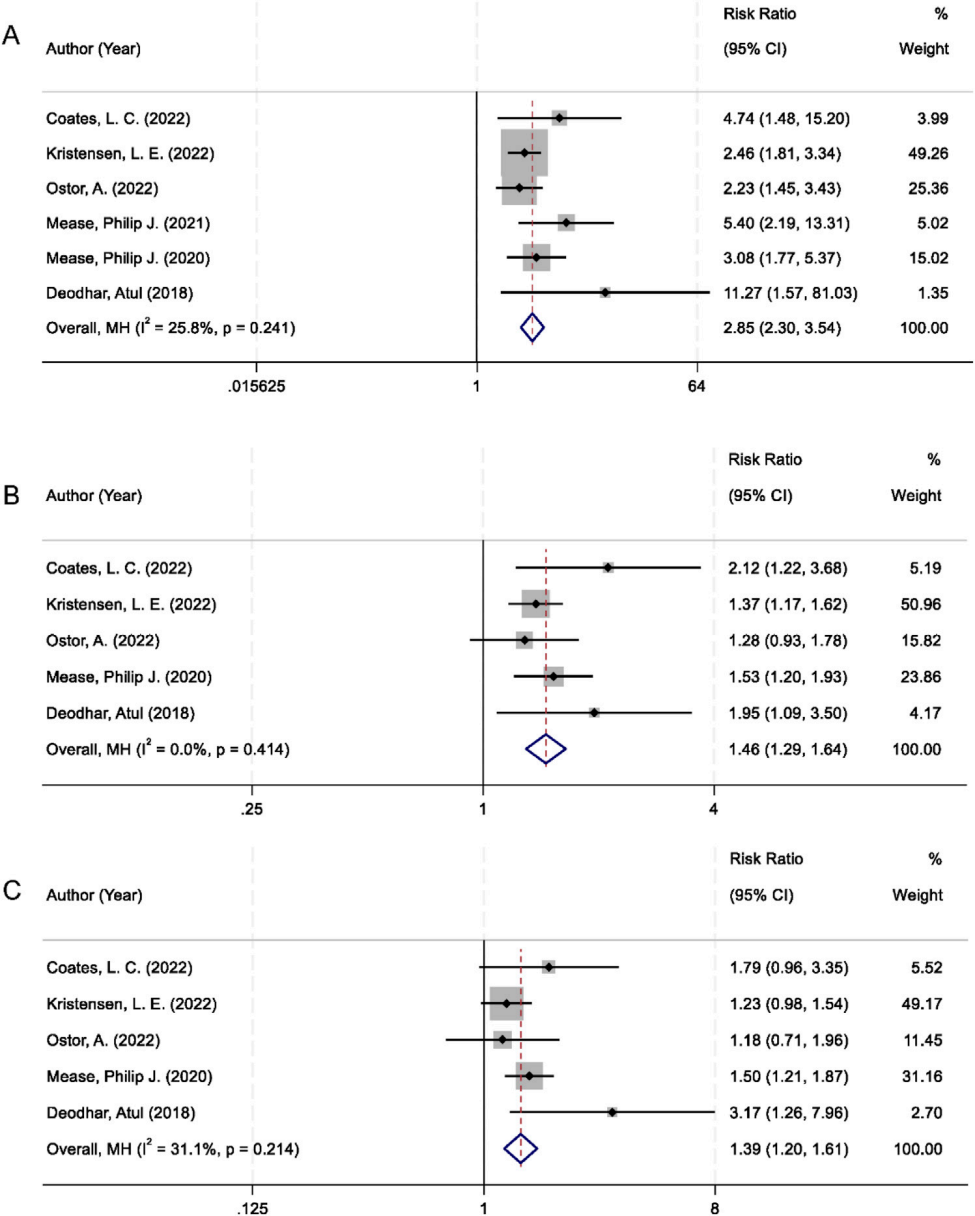
Forest plots comparing IL-23 inhibitors and placebo for: **(A)** Minimal Disease Activity; **(B)** Enthesitis resolution; **(C)** Dactylitis resolution.

Achieving minimal disease activity (MDA) is recognized as a key therapeutic target in the management of PsA reflecting comprehensive control of both articular and extra-articular manifestations. All six randomized controlled trials included in the meta-analysis reported MDA as an outcome. The pooled analysis demonstrated that patients treated with IL-23 inhibitors had a significantly higher MDA response rate compared to those receiving placebo. The combined RR was 2.85 (95% CI 2.30–3.54; p < 0.001), with low heterogeneity among studies (I^2^ = 25.8%; Figure 4A).

In addition to global disease activity, the resolution of specific musculoskeletal manifestations, such as enthesitis and dactylitis, was also assessed. These clinical features are not only prevalent in PsA but are also indicative of disease severity and functional impairment. Data from five trials were available for both endpoints. Patients receiving IL-23 inhibitors exhibited a significantly greater resolution of enthesitis compared to placebo (RR = 1.46; 95% CI: 1.29–1.64; p < 0.001), with no evidence of heterogeneity (I^2^ = 0.0%; Figure 4B). Similarly, a significant improvement in dactylitis resolution was observed in the IL-23 inhibitor group (RR = 1.39; 95% CI: 1.20–1.61; p < 0.001), with moderate heterogeneity (I^2^ = 31.1%; Figure 4C).

### 3.6 Assessment of publication bias

To evaluate the potential for publication bias, Egger’s linear regression test was conducted across all included outcome measures. The results demonstrated no statistically significant evidence of small-study effects or publication bias (all p-values >0.05). This suggests that the findings of the meta-analysis are unlikely to be influenced by selective reporting or the preferential publication of positive results, thereby reinforcing the reliability and internal validity of the pooled estimates.

## 4 Discussion

The findings from this meta-analysis provide robust evidence supporting the clinical efficacy of IL-23 inhibitors in the treatment of psoriatic arthritis (PsA). The analysis demonstrated significantly higher response rates for ACR20, ACR50, and ACR70 among patients receiving IL-23 inhibitors compared to those receiving placebo. The lack of significant heterogeneity across studies further strengthens the reliability of these results. Additionally, IL-23 inhibitors yielded a markedly superior PASI90 response, highlighting their therapeutic benefit not only in joint-related outcomes but also in skin lesion control. IL-23 plays a central role in the pathophysiology of PsA by promoting the differentiation and maintenance of Th17 cells, which are critical drivers of inflammation in both synovial and cutaneous tissues (Thatiparthi et al., 2021; Alunno et al., 2015). Elevated levels of IL-23 have been identified in the synovium and entheses of PsA patients, where it contributes to the upregulation of pro-inflammatory cytokines such as IL-17 and IL-22. These downstream mediators are implicated in keratinocyte hyperproliferation, neutrophil recruitment, osteoclastogenesis, and bone remodeling, which are hallmark features of PsA (Gialouri et al., 2022). By inhibiting IL-23, this inflammatory cascade is interrupted, resulting in clinical improvements across multiple disease domains. Moreover, the analysis demonstrated that IL-23 inhibitors significantly increased the proportion of patients achieving MDA, a comprehensive therapeutic target that reflects optimal control of both articular and extra-articular manifestations. In addition to overall disease control, IL-23 inhibition was associated with significant improvements in enthesitis and dactylitis, which are key clinical features indicative of more severe disease and functional impairment. These results suggest that IL-23 blockade may effectively target inflammation at both axial and peripheral sites of musculoskeletal involvement, aligning with current mechanistic insights regarding its role in enthesis-resident immune cell activation and bone pathology (Su et al., 2024; Floris et al., 2025).

The present findings are consistent with those reported in pivotal clinical trials and previously published systematic reviews. For instance, randomized controlled trials such as PSUMMIT-1 and PSUMMIT-2 have demonstrated that IL-12/23 and IL-23-specific inhibitors, such as ustekinumab and guselkumab, significantly improve ACR response rates and skin outcomes in PsA. The DISCOVER-1 and DISCOVER-2 trials further established the efficacy of guselkumab in both biologic-naïve and biologic-experienced patients, confirming improvements in joint and skin domains and high rates of MDA achievement. Furthermore, network meta-analyses have placed IL-23 inhibitors favorably among biologic and targeted synthetic DMARDs in terms of skin efficacy, often outperforming TNF inhibitors and IL-17 inhibitors in PASI response rates (Lee and Moon, 2023; Krueger et al., 2019). While head-to-head comparisons with TNF inhibitors are limited, the overall clinical benefit profile of IL-23 inhibitors, particularly in patients with prominent cutaneous disease or intolerance to other agents, supports their utility as a viable therapeutic option. Mechanistically, IL-23 inhibitors provide a more targeted immunomodulatory effect compared to IL-12/23 dual blockade (Mease et al., 2023). Recent studies suggest that IL-12 may possess protective anti-inflammatory functions, and its inhibition could attenuate the overall therapeutic efficacy. Therefore, IL-23p19-specific agents may offer superior disease control with a favorable safety profile. Although IL-23 inhibition demonstrated robust efficacy across peripheral and cutaneous domains of PsA, current evidence suggests limited benefit in axial disease. Clinical trials of IL-23 inhibitors in axial spondyloarthritis and axial PsA subgroups have not shown significant improvement in spinal pain or inflammation scores compared with placebo. This may reflect distinct immunopathogenic mechanisms in axial involvement, which appears to be drziven predominantly by IL-17A and TNF-α pathways independent of IL-23 signaling. These findings highlight that IL-23 blockade is most effective for peripheral and entheseal manifestations, whereas its role in axial PsA remains uncertain, underscoring the need for mechanism-tailored therapeutic strategies (Mohanakrishnan et al., 2022; Nerviani et al., 2021).

This meta-analysis provides the most up-to-date synthesis of randomized trials evaluating IL-23 inhibitors in PsA, incorporating data from recent phase III studies to enhance the strength and relevance of the evidence. The findings underscore the clinical applicability of IL-23 inhibition as a targeted strategy for PsA patients, particularly those with moderate-to-severe joint involvement, extensive psoriasis, or inadequate response to existing biologic therapies. IL-23 inhibitors have demonstrated significant improvements in enthesitis, dactylitis, and MDA attainment, supporting their use in patients with complex or refractory disease phenotypes. Given their distinct mechanism of action, IL-23 inhibitors also provide a valuable therapeutic alternative for patients who are contraindicated for TNF or IL-17 inhibitors. Treatment decisions should be guided by the specific clinical manifestations, comorbid conditions, and patient preferences.

The safety profile of IL-23 inhibitors in PsA is generally favorable, with adverse event rates similar to placebo and no new safety signals beyond those observed in psoriasis populations. Common adverse events include nasopharyngitis, upper respiratory tract infections, and injection-site reactions, while transient, asymptomatic elevations in hepatic transaminases are rare and typically do not require treatment discontinuation (Demirel Öğüt et al., 2025; Blauvelt et al., 2023; Huang et al., 2023). No increase in serious infections, malignancy, or cardiovascular events has been noted. Recent studies support the sustained efficacy and safety of IL-23 inhibitors in various clinical settings, including among biologic-experienced patients (Blauvelt et al., 2023; Gooderham et al., 2022). Observational studies suggest favorable drug survival over extended follow-up, with low rates of discontinuation and continued improvements in ACR and PASI scores, further reinforcing the long-term role of IL-23 inhibitors in disease management (Thomas et al., 2024; Borghi et al., 2024). Early intervention studies, such as the Interleukin-23 Inhibition in Very Early Psoriatic Arthritis (IVEPSA) trial, suggest that IL-23 inhibition may delay the onset of PsA in high-risk psoriasis patients by modulating subclinical inflammation, highlighting its potential as a preventive approach (Witte et al., 2025). Furthermore, IL-23 inhibitors appear effective and safe in special populations, including patients with concurrent inflammatory bowel disease, obesity, or advanced age (Blauvelt et al., 2023). Their use has been associated with consistent improvements in patient-reported outcomes, such as enhanced quality of life, reduced fatigue, and better work productivity (Witte et al., 2025). These benefits are further supported by significant improvements in standardized measures, such as the Psoriatic Arthritis Impact of Disease (PsAID) score and the Health Assessment Questionnaire Disability Index (HAQ-DI) (Mease et al., 2011; Queiro et al., 2020). Collectively, these findings highlight the broad, patient-centered benefits of IL-23 inhibitors in managing PsA across diverse clinical scenarios.

Our findings are consistent with and extend prior evidence supporting the efficacy of IL-23 inhibitors in psoriatic arthritis. Gossec et al. (2025) study demonstrated that guselkumab significantly improved composite disease activity indices in patients with prior inadequate response to TNF inhibitors, underscoring its utility in biologic-experienced populations. In contrast, our meta-analysis incorporates a broader patient spectrum, including both biologic-naïve and experienced individuals, and evaluates multiple IL-23 inhibitors, thereby enhancing the external validity and generalizability of the conclusions. Similarly, Ritchlin et al. (2024) study confirmed the durable, multi-domain efficacy of guselkumab over 100 weeks, regardless of baseline characteristics. Our pooled analysis corroborates these long-term benefits while offering a more robust estimate of efficacy across joint, skin, and entheseal domains using aggregated trial-level data. The integrated analysis of the Merola et al. (2024) further validated the sustained efficacy of risankizumab across diverse demographic and clinical subgroups. Our results align with this evidence, while also reinforcing the broader class effect of IL-23 inhibition by evaluating multiple agents and endpoints, such as ACR20/50/70, PASI90, MDA, and resolution of dactylitis and enthesitis. Moreover, our study includes more recent data not captured in the earlier meta-analysis by Vahidy et al. (2023), which reported efficacy of IL-23 inhibitors, particularly guselkumab and ustekinumab, in improving ACR and PASI responses. Unlike that study, our analysis emphasizes the comparative effectiveness of IL-23 inhibitors across newer agents, includes detailed safety data, and highlights their value in refractory and complex PsA phenotypes. Collectively, our findings strengthen the clinical rationale for IL-23 inhibitors as a targeted and versatile treatment option in PsA, while addressing prior knowledge gaps through updated and comprehensive quantitative synthesis.

This meta-analysis has several strengths. It included only randomized controlled trials, providing high-quality evidence. The pooled estimates were robust and demonstrated minimal heterogeneity, suggesting consistency of outcomes across diverse study populations and IL-23 inhibitor subtypes. Multiple domains of disease activity were evaluated, including joint, skin, and enthesis involvement, offering a comprehensive assessment of treatment efficacy. Furthermore, the analysis included evaluation of composite indices such as MDA, which align with treat-to-target strategies in PsA. Several limitations should be acknowledged. First, heterogeneity across studies is inherent due to variability in trial designs, patient populations, IL-23 inhibitors used, dosing regimens, and disease severity (e.g., biologic-naïve vs. biologic-experienced). Although statistical tests for heterogeneity were performed, this variability must be considered when interpreting the overall treatment effect. Secondly, most studies included in this meta-analysis had follow-up periods of 1–2 years, during which IL-23 inhibitors demonstrated robust short-to medium-term efficacy. However, to fully understand the long-term durability of these benefits, particularly in terms of sustained disease control and safety, further extended follow-up studies are needed. The potential for bias in safety reporting also exists, as adverse event reporting may be underrepresented in trials with smaller sample sizes or shorter follow-ups. Additionally, the exclusion of patients with significant comorbidities could lead to an underestimation of safety concerns in real-world populations. Further, rare adverse events such as severe hepatic toxicity or malignancies were not adequately addressed. While common adverse events were well-documented, long-term observational studies and real-world data are essential to capture these less frequent but potentially serious risks. Additionally, there is a lack of data in certain subgroups (e.g., elderly patients, those with comorbidities like inflammatory bowel disease, or severe forms of PsA), highlighting the need for subgroup analyses to assess the efficacy and safety of IL-23 inhibitors in these populations. Finally, head-to-head comparisons between IL-23 inhibitors and other biologics (e.g., TNF inhibitors, IL-17 inhibitors) are limited, and such studies are needed to better define the relative effectiveness and safety of these treatments. Future research should focus on long-term safety monitoring, subgroup efficacy, and direct comparisons between biologic classes to optimize treatment strategies for PsA.

## 5 Conclusion

Based on the current meta-analysis, IL-23 inhibitors demonstrate significant efficacy in improving joint and skin symptoms, achieving minimal disease activity, and resolving enthesitis and dactylitis in patients with psoriatic arthritis. These findings support their integration into treatment strategies, particularly for individuals with multidomain involvement or inadequate response to conventional therapies. Further long-term comparative studies are warranted.

## Data Availability

The raw data supporting the conclusions of this article will be made available by the authors, without undue reservation.
