# Supplementary material for: Clinical benefits and complication profile of IL-23 inhibitors in patients with psoriatic arthritis: a systematic review and meta-analysis
Source: Front Pharmacol. 2025 Nov 6;16:1669786. doi: 10.3389/fphar.2025.1669786 (PMC12631225; doi:10.3389/fphar.2025.1669786)
Supplement: Supplementary file 1 [file Table1.docx]

**Supplementary Table 1 Search Strategy for Four Databases**

| **Database** | **Search Strategy** |
| --- | --- |
| PubMed | ("Psoriatic Arthritis"[Mesh] OR "psoriatic arthritis"[tiab] OR "PsA"[tiab])  AND  ("Interleukin-23"[Mesh] OR "IL-23"[tiab] OR "interleukin 23"[tiab] OR "IL23 inhibitor"[tiab] OR "IL-23 inhibitor"[tiab] OR "guselkumab"[tiab] OR "risankizumab"[tiab] OR "tildrakizumab"[tiab])  AND  ("Randomized Controlled Trial"[Publication Type] OR "randomized controlled trial"[tiab] OR "randomised controlled trial"[tiab] OR "RCT"[tiab]) |
| Embase | ('psoriatic arthritis'/exp OR 'psoriatic arthritis':ti,ab OR 'PsA':ti,ab)  AND  ('interleukin 23'/exp OR 'interleukin 23':ti,ab OR 'IL-23':ti,ab OR 'IL23':ti,ab OR 'IL-23 inhibitor':ti,ab OR 'guselkumab':ti,ab OR 'risankizumab':ti,ab OR 'tildrakizumab':ti,ab)  AND  ('randomized controlled trial'/exp OR 'randomized controlled trial':ti,ab OR 'randomised controlled trial':ti,ab OR 'RCT':ti,ab) |
| Web of Science | TS=("psoriatic arthritis" OR "PsA")  AND  TS=("IL-23" OR "interleukin 23" OR "IL-23 inhibitor" OR "IL23 inhibitor" OR "guselkumab" OR "risankizumab" OR "tildrakizumab")  AND  TS=("randomized controlled trial" OR "randomised controlled trial" OR "RCT") |
| Cochrane Library | (psoriatic arthritis OR PsA)  AND  ("IL-23" OR "interleukin 23" OR "IL-23 inhibitor" OR "IL23 inhibitor" OR guselkumab OR risankizumab OR tildrakizumab)  AND  ("randomized controlled trial" OR RCT) |
